# Safety and Efficacy of Percutaneous Ventricular Assist Device vs Intra-Aortic Balloon Pump in Elective High-Risk Percutaneous Coronary Intervention Procedures

**DOI:** 10.1016/j.jscai.2025.103997

**Published:** 2025-11-04

**Authors:** Tayyab Shah, Chantal Holy, Ali Almedhychy, Jeffrey W. Moses, Helen Parise, Alejandro Lemor, William W. O’Neill, Alexandra J. Lansky

**Affiliations:** a Yale Cardiovascular Research Group, Yale School of Medicine, New Haven, Connecticut; b Division of Cardiovascular Medicine, Hospital of the University of Pennsylvania, Philadelphia, Pennsylvania; c Johnson & Johnson, Co, New Brunswick, New Jersey; d Johnson & Johnson Co., Danvers, Massachusetts; e NewYork-Presbyterian Hospital/Columbia University Irving Medical Center, New York, New York; f St. Francis Heart Center, Roslyn, New York; g University of Mississippi Medical Center, Jackson, Mississippi; h Henry Ford Hospital System, Detroit, Michigan

**Keywords:** high-risk percutaneous coronary intervention, intra-aortic balloon pump, mortality rate, percutaneous ventricular assist device, propensity score matching

## Abstract

**Background:**

This study compares outcomes between percutaneous ventricular assist device (PVAD)-supported and intra-aortic balloon pump (IABP)-supported high-risk percutaneous coronary intervention (HRPCI) in a large-scale, contemporary hospital administrative dataset.

**Methods:**

Patients undergoing HRPCI supported by PVAD or IABP between January 1, 2018, and April 30, 2024, were identified in the Premier Healthcare Database. Patients were excluded if they had cardiogenic shock and/or STEMI on admission, received mechanical circulatory support and PCI on different days, required emergent procedures, had multiple mechanical circulatory support devices used, or received coronary artery bypass grafting surgery within the same admission. Variable rate propensity score matching was conducted with 87 preprocedural variables including patient demographic characteristics, comorbidities, prior procedures, prior complications, and provider/hospital factors. The primary end point was the 90-day mortality, and secondary end points included major adverse cardiac and cerebrovascular events (defined as death, myocardial infarction, or stroke), new cardiogenic shock, 30-day acute kidney injury, in-hospital blood transfusions, length of stay, and discharge disposition.

**Results:**

A total of 2416 patients who underwent PVAD-assisted HRPCI and 847 patients who underwent IABP-assisted HRPCI (mean age, 72 ± 10.4 years; 66% women; 19% non-White) were matched with good balance among all matched variables. Compared with those who received IABP support, those who received PVAD support had lower 90-day mortality (7.9% vs 11.8%; *P* = .01), lower 90-day major adverse cardiac and cerebrovascular events (10.3% vs 14.9%; *P* < .001), less postprocedural cardiogenic shock (9.5% vs 23.5%; *P* < .001), and less acute kidney injury (9.9% vs 15.6%; *P* < .001). PVAD-supported patients had shorter mean lengths of stay (4.3 vs 5.7 days; *P* < .001) and were more likely to be discharged to home. There were no significant differences in rates of blood transfusions (11.3% vs 10.3%; *P* = .43) or vascular complications between groups.

**Conclusions:**

This retrospective observational study suggests that PVAD-supported HRPCI in nonshock patients may be associated with improved clinical outcomes compared with IABP-supported HRPCI; however, future randomized trials are required to confirm this finding.

## Introduction

In patients with high-risk lesions, comorbidities, or compromised hemodynamics, use of mechanical circulatory support (MCS) during high**-**risk percutaneous coronary intervention (HRPCI) can mitigate hemodynamic instability during procedures and allow for more complete revascularization.^1^, ^2^, ^3^, ^4^, ^5^, ^6^, ^7^ Percutaneous ventricular assist devices (PVAD; eg, Impella; Abiomed) and intra-aortic balloon pumps (IABP) are commonly used in this setting, with most supported HRPCI cases using PVAD in contemporary practice.^3^ The PROTECT II randomized controlled trial compared routine IABP support vs routine PVAD support for HRPCI and found that although PVAD provided superior hemodynamic support and resulted in more complete revascularization, there was no difference in 30-day major adverse events, and there were more bleeding and vascular complications with PVAD.^6^^,^^7^ However, there were trends for improved outcomes with PVAD at 90 days. Over the decade since PROTECT II, there have been significant improvements in both the PVAD devices and operator experience, necessitating updated comparisons.^2^

Various observational studies comparing PVAD-supported versus IABP-supported HRPCI have had conflicting results.^3^^,^^8^, ^9^, ^10^ These mixed results may, in part, be due to inherent limitations of payor databases with the inclusion of mixed patient populations (with and without cardiogenic shock at time of admission), inability to accurately adjust for confounders, the use of data predating contemporary best practices (eg, regarding vascular access and patient selection),^2^^,^^11^^,^^12^ or small sample sizes unsuitable for statistical comparison. The objective of this study is to compare the safety and effectiveness of elective (nonshock) HRPCI supported by either PVAD or IABP using a contemporary large-scale hospital database.

## Materials and methods

### Data source

The Premier Healthcare Database (PHD) contains complete clinical records, including diagnosis, procedures, and hospital-prescribed medications from approximately 25% of all inpatient hospital admissions throughout the United States (>1000 hospitals and hospital systems). The PHD collects data from participating hospitals in its health care alliance. Although the database excludes federally funded hospitals (eg, Veterans Affairs), the hospitals included are nationally representative based on bed number, geographic region, location (urban/rural), and teaching hospital status. The use of the PHD was reviewed by the New England Institutional Review Board and was determined to be exempt, as all patient data in this database are deidentified. While we cannot share data from PHD, it is a database available to the public for purchase, and the code for all statistical analyses can be made available to interested parties upon reasonable request.

### Patient selection

This is a retrospective cohort analysis using patient data from January 1, 2018, through April 30, 2024. Patient records were included if they passed internal quality and validity checks by PHD (determination made by PHD based on their own criteria and not chosen by the authors of this study). Patients were included if they presented for elective percutaneous coronary intervention (PCI; PHD classifies admissions as elective vs urgent/emergent) and received PVAD or IABP support on the same day as PCI. For patients who had multiple hospitalizations with MCS-assisted PCI procedures during the study period, only the first hospitalization was included to avoid duplicate entry of the same patient. A small number of patients equally distributed between the 2 groups (<2%) received multiple PCI procedures during the index admission and were included, with follow-up starting with the first PCI (with all PCI characteristics presented related to first procedure only). Patients were excluded if they had urgent or emergent admissions or had cardiogenic shock and/or acute ST-elevation myocardial infarction present on admission. They were also excluded if they received both IABP and PVAD during the index admission, received a different MCS device, or underwent coronary artery bypass grafting (CABG) during the index admission.

Elective, nonshock patients who underwent PCI and received MCS on the same day were assumed to qualify as undergoing HRPCI based on contemporary definitions rather than based on individual clinical and anatomic criteria.^1^, ^2^, ^3^, ^4^, ^5^, ^6^, ^7^ All diagnoses and procedures are identified by corresponding International Classification of Diseases, 10th Revision (ICD-10) codes or current procedural terminology 4 codes. Complete code lists are listed in Supplemental Table S1.

### End points

The prespecified primary end point was the 90-day mortality. Mortality was based on discharge status per PHD, which was derived from the encounter’s UB-04 discharge forms. Mortality was defined as one of the following: UB-04 Code 20: expired; UB-04 Code 40: expired at home; UB-04 Code 41: expired in medical facility; or UB-04 Code 42: expired, place unknown.

Secondary outcomes included stroke, acute myocardial infarction (AMI), major adverse cardiac and cerebrovascular events (MACCE; the composite of mortality, stroke, and AMI) assessed at 90 days. Other secondary end points included new cardiogenic shock during hospitalization, acute kidney injury (AKI) during hospitalization and up to 30 days postprocedurally, bleeding up to 30 days postprocedurally, blood transfusions during index hospitalization, and vascular complications during index hospitalization. Mean length of stay and discharge disposition were also analyzed as secondary end points.

Stroke, AMI, cardiogenic shock, AKI, bleeding, and vascular complications were identified based on new ICD-10 diagnoses (Supplemental Table S1) during or after index admission (ie, not present on admission). While there are limitations to coding of “present on admission,” prior studies have demonstrated that this coding is accurate and improves risk model performance.^13^^,^^14^ Blood transfusions were identified based on ICD-10 procedure codes and CPT-4 codes (Supplemental Table S1). Length of stay was predefined in the PHD. Discharge disposition was based on the UB-04 descriptors for the encounter.

### Statistical analysis

Data on preprocedural variables including patient demographic characteristics, hospital characteristics, chronic conditions from 365 days before admission (including all those required to calculate the Elixhauser index, a validated tool that uses ICD-10 codes to predict in-hospital mortality^15^^,^^16^), and acute cardiovascular conditions present on admission were collected. Preprocedural variables affecting more than 1% of patients, with standardized mean differences (SMDs) exceeding 0.01 were identified, collinear variables were excluded, and a final list of 87 variables (Supplemental Table S1) were used to calculate the propensity score using logistic regression. While procedural variables including number of vessels treated by PCI (as reported by site) and concurrent use of intravascular ultrasound (IVUS) and atherectomy were available, these were not included in matching to avoid including postinterventional (ie, MCS) variables in the propensity score. Variable rate propensity score matching without replacement was performed to match patients in the PVAD arm with those in the IABP arm. Postmatch SMDs were analyzed for all covariates with good balance defined as SMD of <0.05. Covariate balance was assessed visually using a Love plot (Supplemental Figure S1).

Categorical variables are presented as counts and percentage, while continuous variables are presented as mean with SD. For mortality and MACCE, Kaplan–Meier curves were plotted and groups were compared using both the 90-day K-M event rates (using log-rank test) and using multivariate Cox regression. For other categorical secondary outcomes, event rates were compared using the χ^2^ test with continuity correction as needed, while continuous outcomes (length of stay) were compared using the Kruskal–Wallis rank sum test. The significance threshold was set at α = .05, and multiple testing was not accounted for in the secondary end points.

We conducted multiple sensitivity analyses to ensure the results were robust. First, to ensure that results were not biased by which sites the procedures were being done, we conducted a generalized estimating equation analysis to account for clustering of outcomes at the hospital level (where each site was its own cluster), and we also tested the impact of only including patients who had their procedure done at a hospital that had performed a supported HRPCI using the other device before that given patient’s procedure (to ensure included sites had access to and experience with both technologies). We also tested including patients who underwent CABG during the index admission. All analyses were conducted using R 4.2.2 and RStudio (2024.04.1 Build 748).

## Results

### Patient characteristics

**Figure 1:**
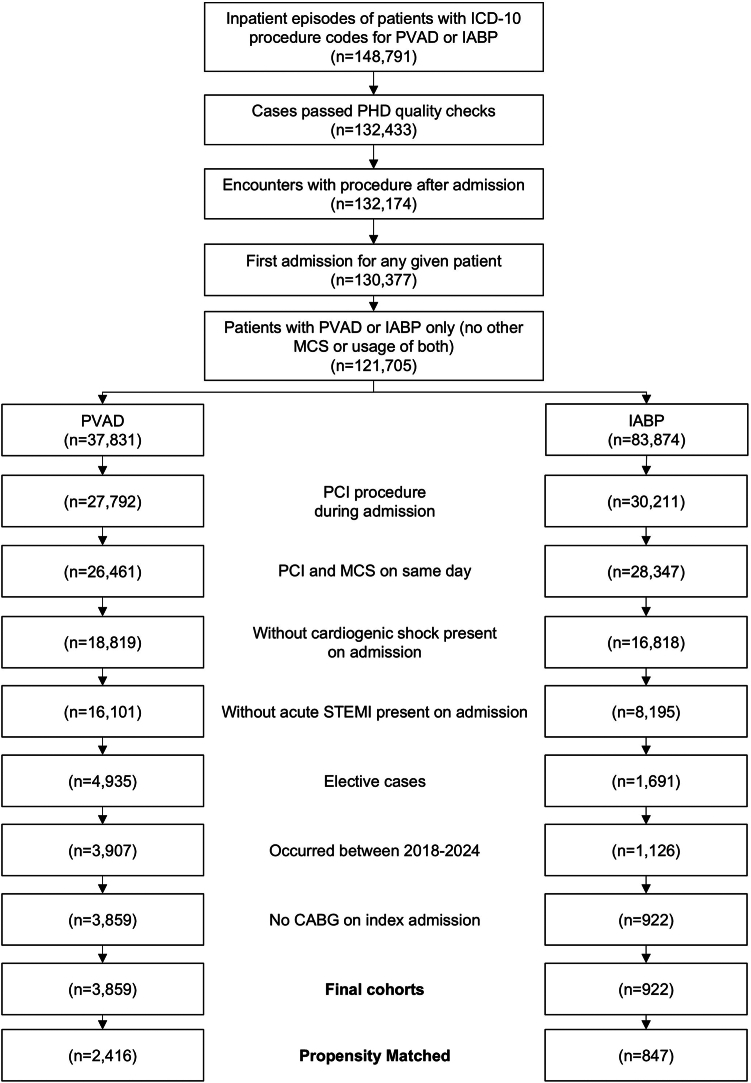
**Attrition flow chart for study cohort selection.** CABG, coronary artery bypass grafting; IABP, intra-aortic balloon pump; MCS, mechanical circulatory support; PHD, Premier Healthcare Database; PVAD, percutaneous ventricular assist device.

Of 148,791 patients identified in the PHD as having PVAD-supported or IABP-supported procedures, a total of 4781 met all inclusion/exclusion criteria (3859 PVAD and 922 IABP) (Figure 1). Before matching, PVAD-supported patients were older (mean age, 73 ± 10.5 vs 71 ± 10.2 years), were more often male (73% vs 64%), and had more chronic comorbidities as measured by the Elixhauser index score (mean score, 5.3 vs 5.0). PVAD-supported patients had higher rates of diabetes (53 % vs 48%), pulmonary circulation disorders (18% vs 13%), and peripheral vascular disorders (33% vs 28%). Patients in the PVAD arm also had higher rates of congestive heart failure (75% vs 59%) and any valvular disease (39% vs 32%) (Supplemental Tables S2-S4 for prematch data).

In total, 847 IABP-supported patients were matched to 2416 PVAD-supported patients. Covariate balance (SMD < 0.05) was achieved for all 87 independent matching variables (Supplemental Figure S1). Residual imbalance for variables not part of the propensity score matching included race and insurance type (Table 1). After matching, the distribution of propensity scores between the IABP and PVAD cohorts is shown in Supplemental Figure S2. The descriptions of patient characteristics and diagnoses after matching are shown in Table 2.

**Table 1:** Patient demographics in matched cohort

Characteristic	PVAD (n =2416)	IABP (n = 847)	SMD
Age, y	72.23 ± 10.45	71.67 ± 10.24	0.054
Age category			0.056
18-34	4 (0.2)	2 (0.2)	
35-44	22 (0.9)	6 (0.7)	
45-54	96 (4.0)	38 (4.5)	
55-64	404 (16.7)	154 (18.2)	
65-74	841 (34.8)	282 (33.3)	
≥75	1049 (43.4)	365 (43.1)	
Male sex	1588 (65.7)	550 (64.9)	0.017
Race			0.127
Asian	55 (2.3)	22 (2.6)	
Black	151 (6.2)	55 (6.5)	
Other	153 (6.3)	68 (8.0)	
Unknown	81 (3.4)	14 (1.7)	
White	1976 (81.8)	688 (81.2)	
Marital status			0.048
Married	1302 (53.9)	474 (56.0)	
Other	152 (6.3)	47 (5.5)	
Single	954 (39.5)	324 (38.3)	
Unknown	8 (0.3)	2 (0.2)	
Payor category			0.124
Commercial	302 (12.5)	142 (16.8)	
Medicaid	128 (5.3)	42 (5.0)	
Medicare	1891 (78.3)	627 (74.0)	
Other	95 (4.0)	36 (4.3)	
Admission year			0.049
2018	472 (19.5)	159 (18.8)	
2019	510 (21.1)	177 (20.9)	
2020	381 (15.8)	135 (15.9)	
2021	389 (16.1)	129 (15.2)	
2022	339 (14.0)	122 (14.4)	
2023	320 (13.3)	124 (14.6)	
2024	3 (0.1)	1 (0.1)	
Urban location	2212 (91.6)	779 (92.0)	0.015
Provider region			0.021
Midwest	703 (29.1)	248 (29.3)	
Northeast	468 (19.4)	164 (19.4)	
South	935 (38.7)	332 (39.2)	
West	310 (12.8)	103 (12.2)	
Hospital size (bed count)			0.037
0-99	42 (1.7)	15 (1.8)	
100-199	167 (6.9)	54 (6.4)	
200-299	322 (13.3)	111 (13.1)	
300-399	403 (16.7)	151 (17.8)	
400-499	308 (12.8)	105 (12.4)	
500+	1173 (48.6)	411 (48.5)	
Teaching hospital	1555 (64.4)	541 (63.9)	0.010

Values are mean ± SD or n (%).

IABP, intra-aortic balloon pump; PVAD, percutaneous ventricular assist device; SMD, standardized mean difference.

**Table 2:** Patient comorbidities in matched cohort

Diagnosis	PVAD (n = 2416)	IABP (n = 847)	SMD
Elixhauser index score	5.06 ± 2.50	4.99 ± 2.43	0.028
Elixhauser index category			0.041
0	28 (1.1)	12 (1.4)	
1-2	342 (14.2)	125 (14.8)	
3-4	695 (28.7)	251 (29.6)	
5+	1351 (55.9)	459 (54.2)	
Noncardiovascular diagnoses			
Pulmonary circulation disorders	322 (13.3)	116 (13.7)	0.011
Peripheral vascular disorders	714 (29.6)	243 (28.7)	0.019
Other neurological disorders	178 (7.4)	59 (7.0)	0.016
Chronic pulmonary disease	628 (26.0)	219 (25.9)	0.003
Renal failure	860 (35.6)	294 (34.7)	0.019
Liver disease	142 (5.9)	49 (5.8)	0.004
Peptic ulcer disease excluding bleeding	17 (0.7)	5 (0.6)	0.014
Rheumatoid arthritis/collagen vascular diseases	86 (3.6)	31 (3.7)	0.005
Coagulopathy	151 (6.2)	52 (6.1)	0.004
Obesity	645 (26.7)	221 (26.1)	0.013
Fluid and electrolyte disorders	497 (20.6)	171 (20.2)	0.010
Blood loss anemia	25 (1.0)	8 (0.9)	0.009
Deficiency anemia	161 (6.7)	52 (6.1)	0.022
Alcohol abuse	47 (1.9)	17 (2.0)	0.006
Drug abuse	53 (2.2)	18 (2.1)	0.005
Psychoses	10 (0.4)	3 (0.4)	0.011
Diabetes	1183 (48.9)	405 (47.8)	0.023
Hypertension	2230 (92.3)	781 (92.2)	0.003
Cancer	134 (5.5)	57 (6.7)	0.050
Cardiovascular diagnoses			
Congestive heart failure	1501 (62.1)	515 (60.8)	0.027
Cardiac arrhythmia	962 (39.8)	329 (38.8)	0.020
Valvular disease (any)	817 (33.8)	285 (33.6)	0.003
Angina	49 (2.0)	17 (2.0)	0.003
Pacemaker present on index	143 (5.9)	49 (5.8)	0.005
Implantable cardioverter defibrillator present	99 (4.1)	33 (3.9)	0.011

Values are mean ± SD or n (%).

IABP, intra-aortic balloon pump; PVAD, percutaneous ventricular assist device; SMD, standardized mean difference.

### Procedural characteristics

In the matched cohorts, patients treated with PVAD were more likely to have HRPCI on 2 (39.5% vs 27.3%), 3 (17.5% vs 9.6%), or ≥4 (4.8% vs 1.7%) arteries than patients treated with IABP, who primarily received PCI to 1 artery (Table 3). PVAD-supported patients were also more likely to undergo any atherectomy (35% vs 24%) and multivessel atherectomy than patients who received IABP support. Finally, PVAD-supported patients were more likely to have IVUS guidance during PCI than IABP-supported patients (37% vs 29%).

**Table 3:** Procedural characteristics in matched cohort

Procedure	PVAD (n = 2416)	IABP (n = 847)	P
PCI: arteries treated			
1	1178 (48.8)	601 (71.0)	<.001
2	955 (39.5)	231 (27.3)	<.001
3	423 (17.5)	81 (9.6)	<.001
≥4	115 (4.8)	14 (1.7)	<.001
Atherectomy			
Total patients treated	847 (35.1)	207 (24.4)	<.001
1 artery	537 (22.2, 63.4)	174 (20.5, 84.1)	<.001^a^
2 arteries	244 (10.1, 28.8)	24 (2.8, 11.6)	<.001^a^
3 arteries	58 (2.4, 6.8)	8 (0.9, 3.9)	.1219
≥4 arteries	8 (0.3, 0.9)	1 (0.1, 0.5)	.5678
Intravascular ultrasound	894 (37.0)	248 (29.3)	<.001

Values are n (%) or n (% of entire cohort, % of patients with atherectomy).

IABP, intra-aortic balloon pump; PCI, percutaneous coronary intervention; PVAD, percutaneous ventricular assist device.

a *P* value based on percentage of patients with atherectomy.

### Outcomes

PVAD-supported patients had a lower incidence of the primary end point, 90-day mortality, than IABP-supported patients (7.9% vs 11.8%; *P* = .011) (Table 4; Central Illustration). PVAD-supported patients also had a lower rate of 90-day MACCE (10.3% vs 14.9%; *P* < .001) (Table 4; Central Illustration), as well as new postprocedural cardiogenic shock (9.5% vs 23.5%; *P* < .001). In addition, patients who received PVAD were more likely to be discharged to home (82.1% vs 72.5%; *P* < .001) and had shorter average hospital stays (4.3 days vs 5.7 days; *P* < .001) (Table 4). Results for 90-day mortality similarly favored PVAD when hospital level clustering was adjusted for (odds ratio, 0.64; 95% CI, 0.49-0.84) and when the 40 IABP and 8 PVAD cases done at hospitals without prior usage of the other MCS device were excluded (odds ratio, 0.64; 95% CI, 0.48-0.85) (Supplemental Figure S3). Results were also consistent when patients who received CABG during index admission were included (Supplemental Table S5).

**Table 4:** Outcomes in matched cohort

Outcomes	PVAD (n = 2416)	IABP (n = 847)	P
90-Day mortality	7.9 (6.6-9.2)	11.8 (9.6-14.0)	.011
90-Day MACCE	10.3 (8.8-11.8)	14.9 (12.4-17.4)	.00019
90-Day MI	110 (4.6)	52 (6.2)	.073
90-Day stroke	27 (1.1)	10 (1.2)	.866
3-Day acute kidney injury	240 (9.9)	132 (15.6)	<.001
30-Day cardiovascular-related bleeding	219 (9.1)	85 (10.0)	.433
In-hospital blood transfusion	273 (11.3)	87 (10.3)	.427
In-hospital vascular complications	49 (2.0)	18 (2.1)	.89
In-hospital new cardiogenic shock	230 (9.5)	199 (23.5)	<.001
Length of hospital stay, d	4.30 ± 5.93	5.73 ± 6.39	<.001
Discharge to home or home health organization	1985 (82.1)	614 (72.5)	<.001

Values for 90-d mortality and MACCE are the Kaplan-Meier event rates presented as % (95% CI); for other outcomes, the raw event rates are presented as n (%) or mean ± SD.

IABP, intra-aortic balloon pump; MACCE, major adverse cardiac and cerebrovascular events; MI, myocardial infarction; PVAD, percutaneous ventricular assist device.

**Central Illustration:**
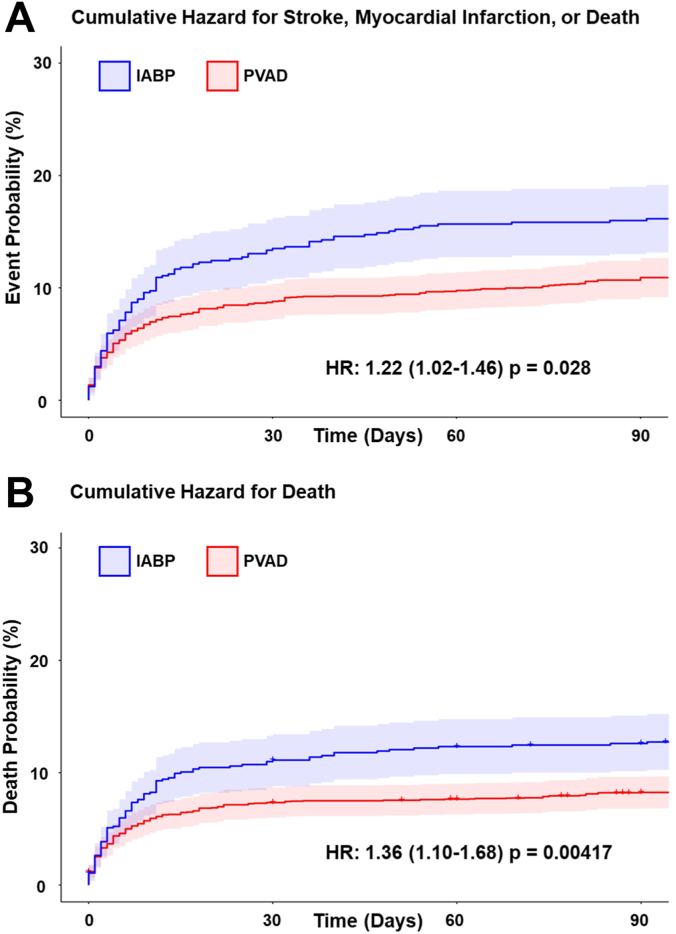
**Kaplan–Meier survival curves for IABP and PVAD.** Cumulative hazard for (**A**) major adverse cardiac and cerebrovascular events (composite of stroke, myocardial infarction, and death); and (**B**) death. HR, hazard ratio; IABP, intra-aortic balloon pump; PVAD, percutaneous ventricular assist device.

Patients who received PVAD support were less likely have an AKI within 30 days of the procedure (9.9% vs 15.6%; *P* < .001). There were no significant differences in rates of procedural complications between the PVAD and IABP groups, with both groups having similar rates of 90-day stroke (1.1% vs 1.2%; *P* = .866), in-hospital transfusions (11.3% vs 10.3%; *P* = .43), 30-day cardiovascular-related bleeding (9.1% vs 10.0%; *P* = .427), and in-hospital vascular complications (2.0% vs 2.1%; *P* = .885) (Table 4).

## Discussion

We report the results of a retrospective observational study comparing PVAD vs IABP support for elective HRPCI in nonshock patients from a large national administrative database. While we used the best available methodology to minimize bias, there are well recognized inherent biases in such comparisons such as the inability to ascertain timing and reason for MCS choice and insertion, numerous unmeasured confounders, coding inaccuracies in the dataset, selection bias in types of patients getting each device in clinical practice, and inadequate ascertainment of secondary end points.^17^ We conducted this study recognizing these limitations, given the lack of adequately powered randomized trials and conflicting data from prior observational studies in this population.^3^^,^^8^, ^9^, ^10^ Our study found that PVAD support was associated with a lower mortality than IABP support without an associated increase in procedure-related adverse events, including stroke, bleeding, or vascular complications. The potential mortality benefit was mostly accrued early, by 30 days postprocedurally. We speculate that it could be related to PVAD allowing for more complete revascularization than IABP (as found in the PROTECT II randomized trial^7^ and consistent with the number of vessels treated in both arms of our study) and/or PVAD potentially preventing hemodynamic instability during the complex procedures—again as suggested by the PROTECT II randomized trial^6^ and consistent with the lower rates of in-hospital cardiogenic shock and 30-day AKI in the PVAD arm of our study. PVAD was also associated with benefits to multiple patient-centered and cost-saving outcomes including hospital length of stay and discharge to home. Still, these results are only hypothesis generating and warrant confirmation in randomized trials such as the ongoing PROTECT IV trial (NCT04763200), which will shed further light on this topic.

To date, there are 2 major MCS trials in the nonshock HRPCI population—BCIS-1 (Balloon Pump–Assisted Coronary Intervention Study), comparing IABP-supported HRPCI with unsupported HRPCI; and the PROTECT II study, comparing PVAD-supported with IABP-supported HRPCI. BCIS-1 did not meet its 28-day primary end point; however, long-term follow-up of the patients in this trial (median, 51 months) found that patients undergoing IABP-supported HRPCI had a 34% lower mortality than patients in the unsupported arm.^5^ The PROTECT II study was also a negative study that did not demonstrate the superiority of PVAD support compared with that of the IABP support. However, there were trends toward improvements in select populations, and at longer follow-up,^6^^,^^18^ this trial used an earlier iteration of PVAD, which provided less support, and there was significantly less experience with safe large-bore access at the time of this trial with complication rates being markedly less in contemporary practice.^11^ Thus, updated analyses and randomized trials are warranted given the risk–benefit equation regarding PVAD has changed substantially since this initial small trial.

There have been a number of observational studies addressing this question since the PROTECT II trial, including multiple prior studies from the PHD database, other administrative databases, and the CathPCI Registry, some of which showed harm with PVAD, while others showed benefit.^3^^,^^8^, ^9^, ^10^ Several aspects of our study set it apart from other published studies and, we believe, may make it less biased, albeit likely still substantially biased. First, some prior studies have included patients with shock on admission (which is a much sicker population less accurately assessed in an administrative database) or excluded patients with shock at any point during admission (which would likely exclude sicker IABP patients who went into shock potentially after inadequate support). While other studies have included patients from 2022 and earlier (and most before 2019), our study includes a large contemporary cohort from 2018-2024 (during which time the Impella CP was available, and operators had gained more experience with larger bore access). Our study also uses a more comprehensive list of variables for matching (2 to 3-fold more than prior analyses), including important variables contributing to high bleed risk criteria (eg, liver disease, prior bleeds, and malignancy)^19^ that have not been used in prior analyses, as well as reporting a more comprehensive list of patient-centered and clinical outcomes (including AKI). Moreover, we adjusted for differential access to IABP/PVAD at different centers which not all prior analyses have done. Finally, unlike prior studies, we did not adjust for procedural variables including number of vessels treated and atherectomy, as these may often be treatments pursued after MCS implantation and adjusting for postinterventional variables could induce substantial bias.

### Limitations

This is an observational study subject to inherent limitations of residual confounding, particularly given the numerous unmeasured characteristics in an administrative dataset, including patient hemodynamics, available access sites, and coronary anatomy. These factors likely affected decision making on MCS choice and subsequent patient outcomes. However, it would be expected that sicker patients would receive the PVAD device (as also observed in this study), thus much of the residual confounding related to unmeasured markers of illness, is likely to disfavor the PVAD arm. It is unusual that higher rates of bleeding and vascular complications were not observed with the larger bore PVAD devices.^6^^,^^20^ Whether this is due to selection bias in which patients received the larger bore device (ie, PVAD-supported patients selected to have adequate vascular access), more experienced operators with more knowledge on managing large bore access choosing PVAD, inadequate ascertainment of the outcomes due to coding errors, or inconsistent reporting across sites and providers cannot be ascertained. It is reassuring, however, that the rates of mortality, MI, stroke, in-hospital transfusions, and vascular complications were similar to those reported by the prospective PROTECT III study.^11^ Notably, this type of selection bias would also be present in a randomized trial comparing the 2 devices, as clinicians would only enroll patients with suitable anatomy for both devices. The rate of in-hospital cardiogenic shock in the IABP group is also unrealistically high (>20%), with prior studies reporting event rates closer to 10%.^3^^,^^6^^,^^21^ This may in part be because coding for cardiogenic shock may be inaccurate, and it may have been present before the procedure for many of these patients. Similarly, the exact timing of MCS device implantation could not be determined with specificity beyond knowing it occurred on the same day as HRPCI. Thus, some patients may have received the device as a bailout strategy due to complications from an elective PCI and not necessarily as a prophylactic measure, which we were aiming to capture. Still, it should be noted that both arms should be affected by this and the relative benefit of PVAD compared with IABP on preventing cardiogenic shock was similar in magnitude to that observed in the PROTECT II trial^6^ and the recent analysis from the CathPCI Registry, which does have more detailed data on these issues.^3^ Finally, it should also be noted that PVAD-supported patients more often received IVUS guidance during PCI, perhaps in the hands of more experienced interventionalists, as guidelines now recommend.^22^^,^^23^ This suggests that patients receiving PVAD received more interventional evidence-based care (and possibly better medical care), which may explain the substantial mortality benefit observed. Finally, while operator experience is known to impact patient outcomes, we were unable to adjust for operator experience.^8^^,^^10^ That said, we did adjust for hospital level clustering, which does account for some of the variation related to operator experience, volume, and skill.

## Conclusions

This observational study suggests that PVAD-assisted HRPCI may be associated with improved postprocedural hemodynamics and 90-day clinical outcomes. However, these findings are hypothesis generating and require confirmation in randomized trials.
