# Supplementary material for: Safety and Efficacy of Percutaneous Ventricular Assist Device vs Intra-Aortic Balloon Pump in Elective High-Risk Percutaneous Coronary Intervention Procedures
Source: J Soc Cardiovasc Angiogr Interv. 2025 Nov 4;4(12):103997. doi: 10.1016/j.jscai.2025.103997 (PMC12766050; doi:10.1016/j.jscai.2025.103997)
Supplement: Supplemental Material [file mmc1.docx]

**Supplemental Material**

**Supplemental Table S1: Variable Definitions**

A: Procedure Code List

B: Diagnoses Code List

C: Final Matching Variables

**Supplemental Table S2: Pre-match Patient Demographics and Provider Characteristics**

| **Variables** | **PVAD** | **IABP** | **SMD** |
| --- | --- | --- | --- |
| n | 3,859 | 922 |  |
| Age (mean (SD)) | 72.85 (10.47) | 71.40 (10.24) | 0.141 |
| Age Category (%) |  |  | 0.148 |
| 18 - 34 | 4 ( 0.1) | 2 ( 0.2) |  |
| 35 - 44 | 32 ( 0.8) | 6 ( 0.7) |  |
| 45 - 54 | 163 ( 4.2) | 44 ( 4.8) |  |
| 55 - 64 | 612 ( 15.9) | 171 ( 18.5) |  |
| 65 - 74 | 1,185 ( 30.7) | 319 ( 34.6) |  |
| 75 Plus | 1,863 ( 48.3) | 380 ( 41.2) |  |
| Gender = Male (%) | 2,796 ( 72.5) | 588 ( 63.8) | 0.187 |
| Race Category (%) |  |  | 0.140 |
| Asian | 105 ( 2.7) | 24 ( 2.6) |  |
| Black | 310 ( 8.0) | 58 ( 6.3) |  |
| Other | 250 ( 6.5) | 73 ( 7.9) |  |
| Unknown | 129 ( 3.3) | 15 ( 1.6) |  |
| White | 3,065 ( 79.4) | 752 ( 81.6) |  |
| Marital Status (%) |  |  | 0.045 |
| Married | 2,079 ( 53.9) | 514 ( 55.7) |  |
| Other | 224 ( 5.8) | 49 ( 5.3) |  |
| Single | 1,543 ( 40.0) | 357 ( 38.7) |  |
| Unknown | 13 ( 0.3) | 2 ( 0.2) |  |
| Payor Category Summary (%) |  |  | 0.166 |
| Commercial | 445 ( 11.5) | 159 ( 17.2) |  |
| Medicaid | 189 ( 4.9) | 45 ( 4.9) |  |
| Medicare | 3,075 ( 79.7) | 680 ( 73.8) |  |
| Other | 150 ( 3.9) | 38 ( 4.1) |  |
| Admission Year (%) |  |  | 0.200 |
| 2018 | 584 ( 15.1) | 181 ( 19.6) |  |
| 2019 | 737 ( 19.1) | 187 ( 20.3) |  |
| 2020 | 530 ( 13.7) | 148 ( 16.1) |  |
| 2021 | 614 ( 15.9) | 140 ( 15.2) |  |
| 2022 | 717 ( 18.6) | 137 ( 14.9) |  |
| 2023 | 648 ( 16.8) | 128 ( 13.9) |  |
| 2024 | 29 ( 0.8) | 1 ( 0.1) |  |
| Urban/Rural = Urban (%) | 3,618 ( 93.8) | 843 ( 91.4) | 0.089 |
| Provider Region (%) |  |  | 0.318 |
| Midwest | 1,101 ( 28.5) | 269 ( 29.2) |  |
| Northeast | 420 ( 10.9) | 200 ( 21.7) |  |
| South | 1,701 ( 44.1) | 344 ( 37.3) |  |
| West | 637 ( 16.5) | 109 ( 11.8) |  |
| Hospital size: by Bed Count (%) |  |  | 0.104 |
| 000-099 | 76 ( 2.0) | 15 ( 1.6) |  |
| 100-199 | 353 ( 9.1) | 60 ( 6.5) |  |
| 200-299 | 486 ( 12.6) | 116 ( 12.6) |  |
| 300-399 | 679 ( 17.6) | 169 ( 18.3) |  |
| 400-499 | 453 ( 11.7) | 112 ( 12.1) |  |
| 500+ | 1,812 ( 47.0) | 450 ( 48.8) |  |
| Teaching Status = YES (%) | 2,212 ( 57.3) | 600 ( 65.1) | 0.160 |

Values are mean ± SD or n (%).

PVAD, percutaneous ventricular assist device; IABP, intra-aortic balloon pump; SMD, standardized mean difference.

**Supplemental Table S3: Pre-Match Baseline Patient Diagnoses**

| **Variables** | **PVAD** | **IABP** | **SMD** |
| --- | --- | --- | --- |
| N | 3,859 | 922 |  |
| Elixhauser Index Score (mean (SD)) | 5.33 (2.47) | 4.96 (2.44) | 0.150 |
| Elixhauser Index Score category(%) |  |  | 0.166 |
| Elixhauser Index = 0 | 27 ( 0.7) | 12 ( 1.3) |  |
| Elixhauser Index: 1 or 2 | 420 (10.9) | 142 (15.4) |  |
| Elixhauser Index: 3 or 4 | 1,083 (28.1) | 272 (29.5) |  |
| Elixhauser Index: 5 or above | 2,329 (60.4) | 496 (53.8) |  |
| **Non-Cardiovascular Diagnoses** |  |  |  |
| Pulmonary Circulation Disorders | 682 (17.7) | 123 (13.3) | 0.120 |
| Peripheral Vascular Disorders | 1,274 (33.0) | 259 (28.1) | 0.107 |
| Other Neurological Disorders | 228 ( 5.9) | 64 ( 6.9) | 0.042 |
| Chronic Pulmonary Disease | 1,051 (27.2) | 236 (25.6) | 0.037 |
| Renal Failure | 1,476 (38.2) | 317 (34.4) | 0.080 |
| Liver Disease | 203 ( 5.3) | 53 ( 5.7) | 0.021 |
| Peptic Ulcer Disease Excluding Bleeding | 30 ( 0.8) | 7 ( 0.8) | 0.002 |
| Rheumatoid Arthritis/Collagen Vascular Disease | 124 ( 3.2) | 32 ( 3.5) | 0.014 |
| Coagulopathy | 246 ( 6.4) | 58 ( 6.3) | 0.003 |
| Obesity | 992 (25.7) | 243 (26.4) | 0.015 |
| Fluid And Electrolyte Disorders | 812 (21.0) | 188 (20.4) | 0.016 |
| Blood Loss Anemia | 68 ( 1.8) | 8 ( 0.9) | 0.079 |
| Deficiency Anemia | 220 ( 5.7) | 58 ( 6.3) | 0.025 |
| Alcohol Abuse | 86 ( 2.2) | 17 ( 1.8) | 0.027 |
| Drug Abuse | 76 ( 2.0) | 21 ( 2.3) | 0.021 |
| Psychoses | 11 ( 0.3) | 3 ( 0.3) | 0.007 |
| Diabetes | 2,051 (53.1) | 438 (47.5) | 0.113 |
| Hypertension | 3,601 (93.3) | 845 (91.6) | 0.063 |
| Cancer | 200 ( 5.2) | 63 ( 6.8) | 0.069 |
| **Cardiovascular Diagnoses** |  |  |  |
| Congestive Heart Failure | 2,879 (74.6) | 548 (59.4) | 0.327 |
| Cardiac Arrhythmia | 1,595 (41.3) | 362 (39.3) | 0.042 |
| Valvular Disease | 1,507 (39.1) | 297 (32.2) | 0.143 |
| Pacemaker Present On Index | 224 ( 5.8) | 52 ( 5.6) | 0.007 |
| Implantable Cardioverter Defibrillator Present | 251 ( 6.5) | 35 ( 3.8) | 0.123 |

Values are mean ± SD or n (%).

PVAD, percutaneous ventricular assist device; IABP, intra-aortic balloon pump; SMD, standardized mean difference.

**Supplemental table S4: Pre-Match Cardiovascular Procedures**

| **Variables** | **PVAD** | **IABP** |
| --- | --- | --- |
| N | 3,859 | 922 |
| PCI: 1 artery | 1,844 (47.8) | 654 (70.9) |
| PCI: 2 arteries | 1,541 (39.9) | 254 (27.5) |
| PCI: 3 arteries | 655 (17.0) | 84 ( 9.1) |
| PCI: 4 or more arteries | 179 ( 4.6) | 17 ( 1.8) |
| Atherectomy One Artery Index | 902 (23.4) | 190 (20.6) |
| Atherectomy Two Arteries Index | 394 (10.2) | 26 ( 2.8) |
| Atherectomy Three Arteries Index | 91 ( 2.4) | 9 ( 1.0) |
| Atherectomy Four Or More Artery Index | 22 ( 0.6) | 1 ( 0.1) |
| Intravascular Ultrasound (IVUS) At Time Of Index | 1,539 (39.9) | 267 (29.0) |

Values are n (%) or n (% of entire cohort, % of patients with atherectomy).

PVAD, percutaneous ventricular assist device; IABP, intra-aortic balloon pump; PCI, percutaneous coronary intervention.

**Supplemental Table S5:** Outcomes in unmatched total population including patients that received CABG during the index admission

| **Variables** | **PVAD**  **(n=3,907)** | **IABP**  **(n=1,126)** | **p** |
| --- | --- | --- | --- |
| Length Of Stay (mean (SD)) | 3.94 (5.40) | 6.92 (7.84) | <0.001 |
| In-hospital mortality n(%) | 205 ( 5.2) | 121 (10.7) | <0.001 |
| In-hospital AKI n(%) | 349 ( 8.9) | 198 (17.6) | <0.001 |
| In-hospital Stroke n (%) | 45 ( 1.2) | 33 ( 2.9) | <0.001 |
| In-hospital cardiogenic shock n(%) | 339 ( 8.7) | 288 (25.6) | <0.001 |

**Supplemental Figure S1: Covariate balance before and after PSM**

**
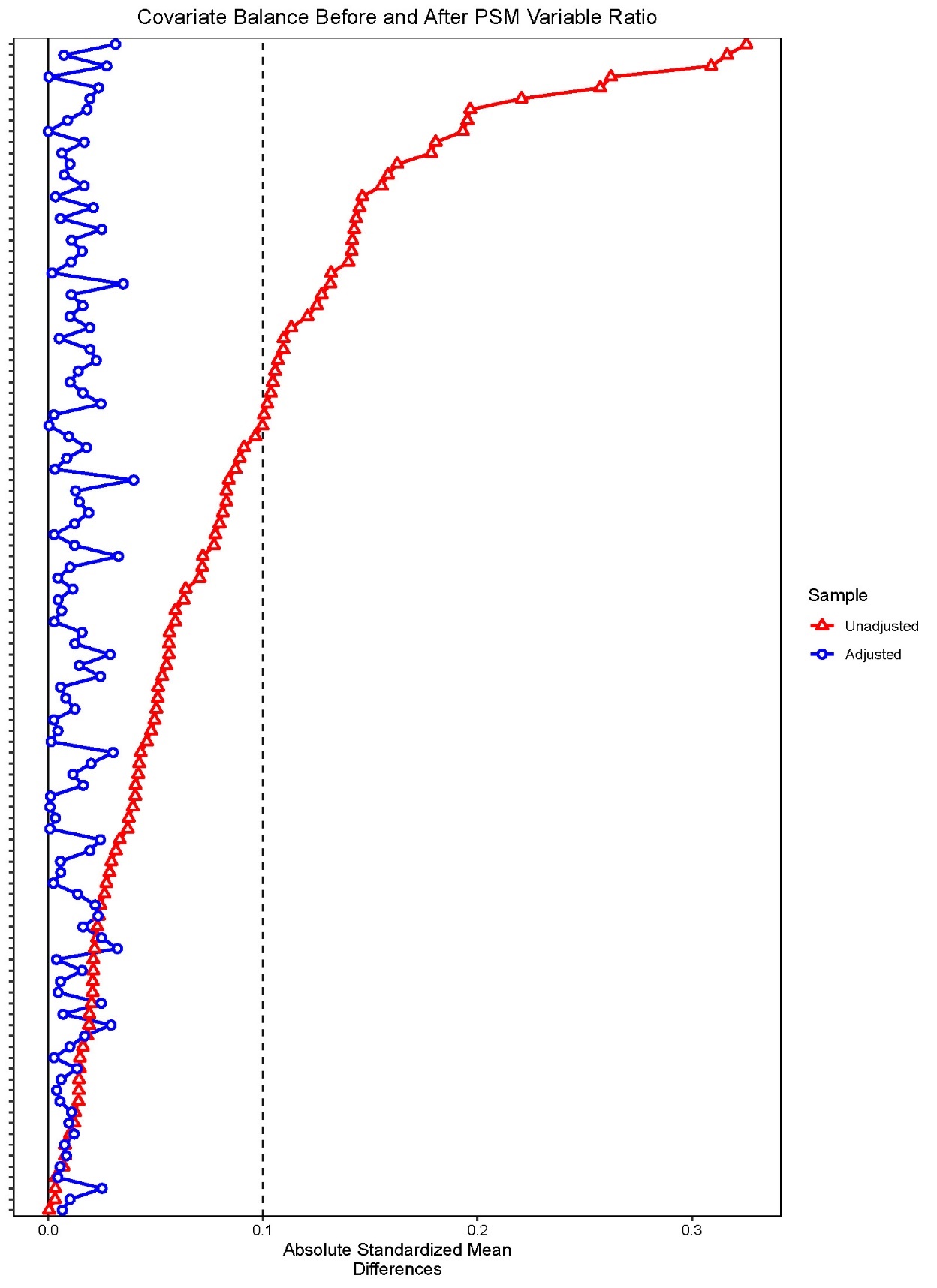
**

**Supplemental Figure S2: Distribution of propensity scores before and after matching.**


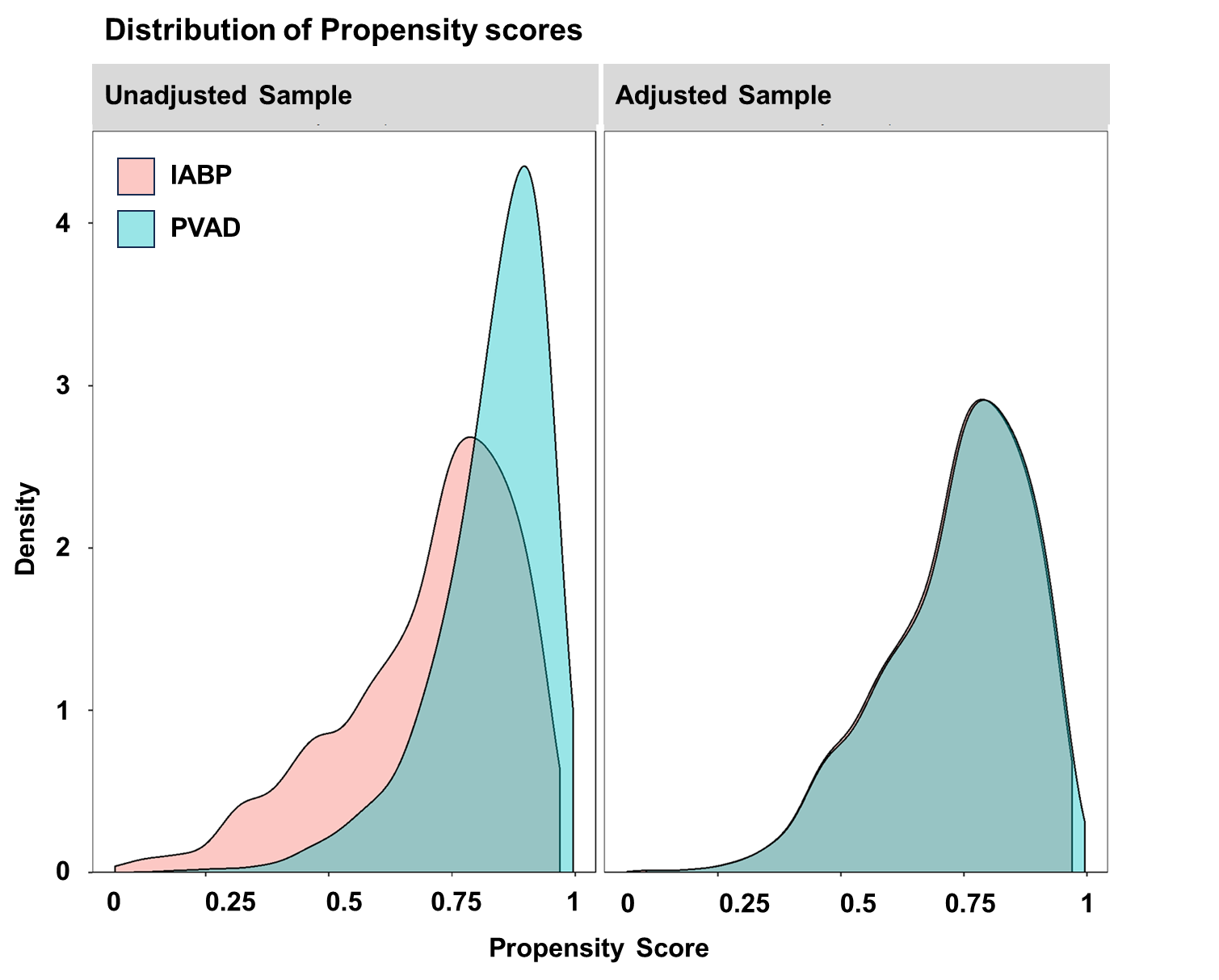


**Supplemental Figure S3: K-M curve for 90-day mortality excluding patients (40 IABP, 8 PVAD) who had procedure done at site without prior usage of the other technology. OR reflects impact of PVAD relative to IABP calculated using GEE models.**

**
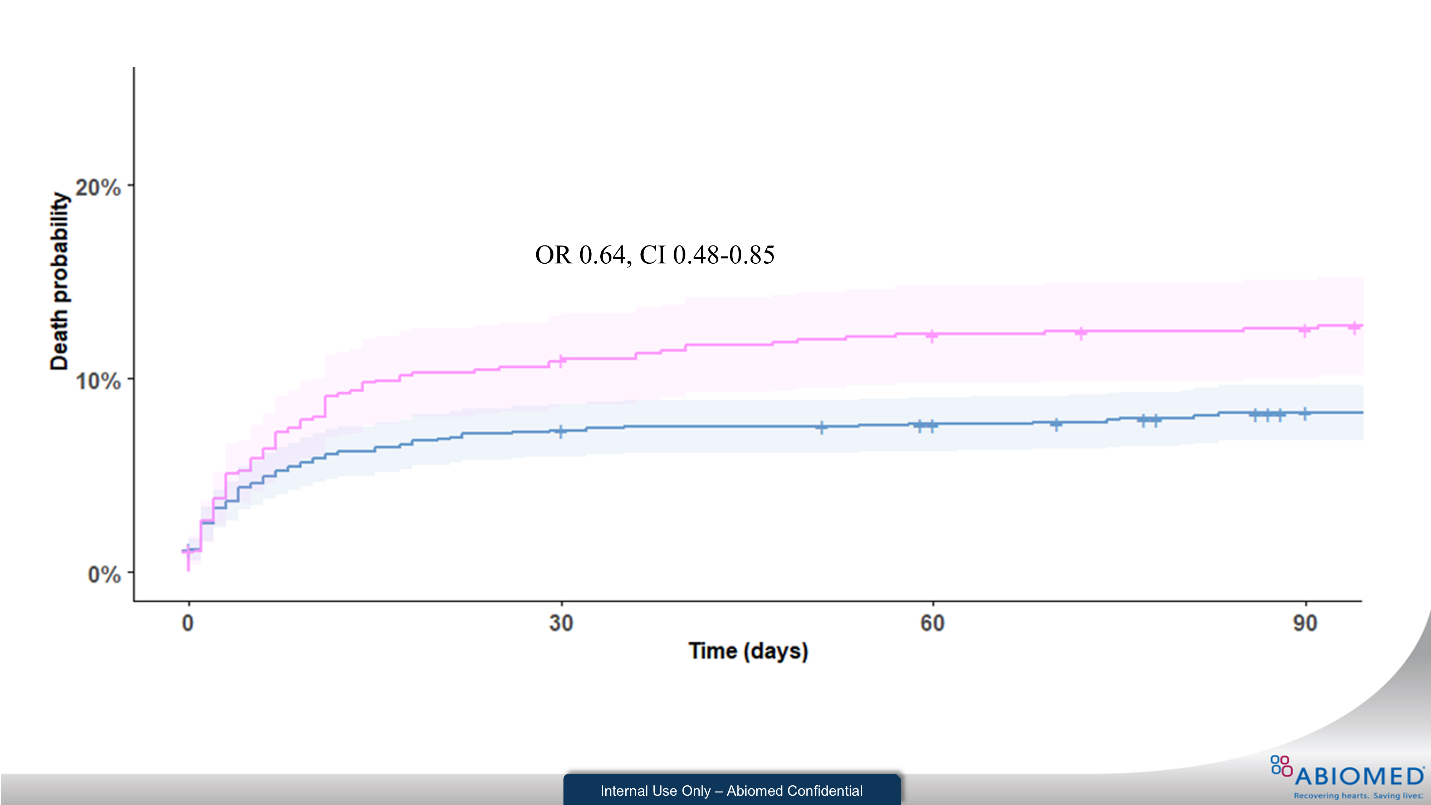
**
